# Posttranslational Modification of Sox11 Regulates RGC Survival and Axon Regeneration

**DOI:** 10.1523/ENEURO.0358-20.2020

**Published:** 2021-02-11

**Authors:** Kun-Che Chang, Minjuan Bian, Xin Xia, Ankush Madaan, Catalina Sun, Qizhao Wang, Liang Li, Michael Nahmou, Takahiko Noro, Satoshi Yokota, Joana Galvao, Alexander Kreymerman, Bogdan Tanasa, Yang Hu, Jeffrey L. Goldberg

**Affiliations:** 1 Spencer Center for Vision Research, Byers Eye Institute, School of Medicine, Stanford University, Palo Alto, CA 94304; 2 Department of Ophthalmology, Louis J. Fox Center for Vision Restoration, University of Pittsburgh School of Medicine, Pittsburgh, Pennsylvania 15213; 3 Department of Ophthalmology, The Jikei University School of Medicine, Tokyo 105-8461, Japan

**Keywords:** optic nerve regeneration, retinal ganglion cell, Sox11, SUMOylation

## Abstract

The failure of adult CNS neurons to survive and regenerate their axons after injury or in neurodegenerative disease remains a major target for basic and clinical neuroscience. Recent data demonstrated in the adult mouse that exogenous expression of Sry-related high-mobility-box 11 (Sox11) promotes optic nerve regeneration after optic nerve injury but exacerbates the death of a subset of retinal ganglion cells (RGCs), α-RGCs. During development, Sox11 is required for RGC differentiation from retinal progenitor cells (RPCs), and we found that mutation of a single residue to prevent SUMOylation at lysine 91 (K91) increased Sox11 nuclear localization and RGC differentiation *in vitro*. Here, we explored whether this Sox11 manipulation similarly has stronger effects on RGC survival and optic nerve regeneration. *In vitro*, we found that non-SUMOylatable Sox11^K91A^ leads to RGC death and suppresses axon outgrowth in primary neurons. We furthermore found that Sox11^K91A^ more strongly promotes axon regeneration but also increases RGC death after optic nerve injury *in vivo* in the adult mouse. RNA sequence (RNA-seq) data showed that Sox11 and Sox11^K91A^ increase the expression of key signaling pathway genes associated with axon growth and regeneration but downregulated *Spp1* and *Opn4* expression in RGC cultures, consistent with negatively regulating the survival of α-RGCs and ipRGCs. Thus, Sox11 and its SUMOylation site at K91 regulate gene expression, survival and axon growth in RGCs, and may be explored further as potential regenerative therapies for optic neuropathy.

## Significance Statement

Sry-related high-mobility-box 11 (Sox11) expression promotes optic nerve regeneration but also increases retinal ganglion cell (RGC) death after optic nerve injury. Here, we demonstrate that mutation of a single SUMOylation site on Sox11 (Sox11^K91A^) leads to stronger effects *in vivo*. RNA sequencing (RNA-seq) analysis reveals that Sox11 and Sox11^K91A^ differentially regulate downstream gene expression related to axon growth and guidance. Understanding these effects of posttranslational modification of Sox11 in regulating regeneration *in vivo* suggests a potent therapeutic strategy for vision restoration in optic neuropathies.

## Introduction

Restoring neuronal connections and function in degenerative diseases and after injury in the CNS is a critical goal for neuroscience and has been investigated for decades, with only a handful of approaches showing promise in preclinical models. In the visual system, retinal ganglion cells (RGCs), projection neurons responsible for transmitting vision from the eye to the brain, have been a major target for research as RGCs undergo cell death and axon degeneration in patients suffering from glaucoma or other optic neuropathies (Goldberg et al., 2016). Thus, optic nerve models are relevant for studying CNS axon regeneration and have pointed to a number of discoveries of signaling pathways implicated in survival and axon regeneration (Benowitz et al., 2017).

A number of transcription factors that play a role in RGC differentiation during early development also have been implicated in regulating survival or axon regeneration in the adult. For example, Pou4f2 contributes to late differentiation of RGCs in the early postnatal period (Badea et al., 2009) and promotes RGC survival in an ocular hypertension model of glaucoma (Stankowska et al., 2015). We and others have shown that Sry-related high-mobility-box4 and 11 (Sox4 an Sox11) are required for RGC differentiation (Jiang et al., 2013; Chang and Hertz, 2017; Chang et al., 2017, 2019; Kuwajima et al., 2017). Increased Sox11 expression has also been shown to promote RGC survival after optic nerve crush (Struebing et al., 2017), and more recently, overexpression of Sox11 was reported to promote axon regeneration but also lead to increased death of α-RGCs (Norsworthy et al., 2017). Thus, more work is required to identify how to target Sox11 for vision restoration while limiting cell death.

Posttranslational modifications such as the addition of the small ubiquitin-related modifier (SUMO) have been show to plays an critical role in regulating Sox11 nuclear localization and gene regulation (Gill, 2005; Roger et al., 2010). We demonstrated that Sox11 SUMOylation at lysine residue 91 (K91) suppressed its nuclear translocation from the cytoplasm; site mutation of Sox11 to prevent SUMOylation led to higher nuclear localization and to an increase in RGC differentiation from retinal progenitor cells (Chang et al., 2017). However, whether Sox11 SUMOylation at K91 is critical for Sox11’s effects on RGC survival, and axon regeneration was still unknown. Here, we studied this question *in vitro* and *in vivo*, and find that as in RGC development, this K91 site is a major regulator of survival and axon growth in the adult. We extend this work using RNA sequencing (RNA-seq) of Sox11-expressing or Sox11^K91A^-expressing RGCs to identify the differentially regulated signaling pathways attributable to Sox11 and Sox11^K91A^. Overall, our results expand on mechanisms of gene regulation contributing to RGC survival and axon regeneration by manipulation of Sox11.

## Materials and Methods

### Animals

All use of animals conformed to the Association for Research in Vision and Ophthalmology (ARVO) Statement for the Use of Animals in Research, and was approved by the Institutional Animal Care and Use Committee (IACUC) and the Institutional Biosafety Committee of Stanford University. C57BL/6 mice both male and female of varying ages were obtained from Charles River.

### Adeno-associated virus (AAV) preparation

Plasmids for Sox11-IRES-EGFP, Sox11^K91A^-IRES-EGFP, and control EGFP were obtained from VectorBuilder. AAV production was performed at the Stanford Vision Research Core (PI, Yang Hu) as previously described (Wang et al., 2020). Briefly, AAV plasmids were co-transfected with pAAV2 (pACG2)-RC triple mutant (Y444, 500, 730F) and pHelper plasmid (Strategene) into human embryonic kidney 293 (HEK 293) cells. The AAVs were purified using 40% polyethylene glycol. The AAV titers were determined by quantitative real-time PCR (qRT-PCR) and diluted to 2 × 10^13^ vector genome (vg)/ml stocks and stored at −80°C until use.

### Cell line cultures and Western blotting

HEK 293 cells were cultured in DMEM medium (Corning) containing fetal bovine serum (FBS; 10%, Invitrogen) and penicillin/streptomycin (PS; 1%, Thermo Fisher Scientific) on six-well tissue culture plates. Cells were manually passaged approximately every 3 d or until ∼80% confluence. Cell lines were kept in 37°C incubation at 5% CO_2_.

For Western blotting, protein samples were collected in Laemmli sample buffer (Thermo Fischer Scientific) and heated to 100°C for 10 min. Proteins were resolved by SDS-PAGE (Bio-Rad) and transferred to PVDF membranes using a semi-dry blotter (Bio-Rad). Membranes were blocked with 5% non-fat milk and then probed with primary antibodies rabbit anti-Sox11 (1:1000, Abcam) or anti-GAPDH (1:2000, Cell Signaling Technology) overnight at 4°C. Membranes were washed and probed with secondary antibodies conjugated to horseradish peroxidase (Millipore), and developed with the Western Blot Substrate kit (Thermo Fischer Scientific) by detecting chemiluminescence using the ChemiDoc XRS+ imaging system (Bio-Rad).

### RGC culture and neurite outgrowth assays

Mouse RGCs from postnatal day (P2) pups were purified by immunopanning using CD90.1 as described previously (Barres et al., 1988). RGCs were plated onto PDL/laminin-coated tissue culture plates in Full Sato (FS) medium including forskolin (5 mm), BDNF (50 ng/ml), and CNTF (10 ng/ml) as described previously (Meyer-Franke et al., 1995). Plating densities were 2.5 × 10^3^ RGCs/well in 24-well plates for neurite outgrowth assay, or 2.5 × 10^5^ RGCs/well in six-well plates for RNA-seq and Western blot assays.

For viral transduction in the neurite outgrowth assay, Sox11 and Sox11^K91A^ AAV2 viruses were diluted to 2 × 10^11^ vg/ml in medium and added at a multiplicity of infection (MOI) of ∼10^5^ vg/cell overnight, and then changed into fresh culture medium for another 2 d. RGCs were immunstained with antibodies against β-III-tubulin. Briefly,equal amount 37°C 4% paraformaldehyde (PFA) was added into the medium for 20 min for fixation. The neurites were stained with mouse anti-β-III-tubulin antibody E7 (1:500, hybridoma from Developmental Studies Hybridoma Bank) overnight at 4°C and probed with Alexa Fluor 647-tagged anti-mouse antibody (1:500; Life technologies) for 2 h at room temperature. GFP^+^ and DAPI^+^ RGCs were imaged on a Zeiss Axio Observer inverted microscope using a 10× objective to measure neurite outgrowth and survival, respectively. Total length of neurites per cell (30–60 cells average per condition per experiment) was measured using ImageJ Simple Neurite Tracer.

### AAV transduction and optic nerve crush *in vivo*

To express Sox11 or Sox11^K91A^ *in vivo*, both eyes of C57BL/6 mice were injected intravitreally with 2 μl of AAV2-luc-EGFP or AAV2-Sox11-EGFP or AAV2-Sox11^K91A^-EGFP at P28, as previous described (Boczek et al., 2019). For optic nerve crush, two weeks after virus injection, the left optic nerve was exposed from outer canthus and pinched for 5 s with a Dumont Fine Science Tools #5 self-closing forceps ∼1.5 mm behind the globe. All optic nerve crush procedures were performed masked to the viral vector treatment. At day 12 after optic nerve crush, 2-μl cholera toxin subunit B (CTB)-conjugated Alexa Fluor 555 (CTB-555, 5 μg/μl; Invitrogen) was intravitreally injected as an anterograde tracer to visualize regenerating axons; animals were euthanized 2 d later, 14 days after crush after optic nerve crush. Optic nerves were dissected and fixed in 4% PFA for 1 h and subsequently washed in PBS. Optic nerves were then incubated in 15% sucrose at 4°C overnight and then in 30% sucrose at 4°C overnight before mounting in Optimal Cutting Temperature mounting medium (Thermo Fisher Scientific) before cutting longitudinal 12-μm-thick cryosections. Optic nerve sections were imaged and analyzed as previously described (Cameron et al., 2020). Briefly, the number of CTB^+^ axons within every 250 μm from the crush site were manually counted to the end of the longest regenerating axons. Total regenerating axons per optic nerve were calculated using the formula as previously described (Bei et al., 2016).

For RGC survival analysis, retinas were dissected and fixed in 4% PFA for 1 h, then permeabilized with 3% Triton X-100 (Sigma) and 1.5% Tween 20 (Sigma) for another hour, blocked with 10% normal goat serum (NGS) in PBS for 1 h, and then incubated with a rabbit polyclonal anti-RBPMS primary antibody (1:500; PhosphoSolution) overnight at 4°C. Retina samples were washed three times, ten minutes each, with PBS and incubated with rabbit polyclonal Alexa Fluor 647-tagged anti-rabbit antibody (1:500; Life technologies) overnight. The explants were then washed twice, ten minutes each, stained with DAPI (1:5000 in PBS) for 15 min, washed twice 10 min each, and sealed under 1.5-mm coverslips with anti-fade mounting medium (ProLong Gold, Life Technologies) before imaging via confocal microscopy (Zeiss). The retinas were divided into four quadrants, and one digital micrograph was taken randomly from each of four peripheral areas 3 mm from the optic nerve head. RBPMS-positive cells were counted manually in a masked fashion and presented as cells per millimeter squared.

### RNA-seq of purified RGCs

For viral transduction of E18 RGCs for RNA-seq, 2.5 × 10^5^ RGC/well were plated on PDL/laminin-coated six-well plates in quadruplicates. Four hours after plating, 1.5-μl AAV (2 × 10^13^ vg/ml) was added to cultured cells at a MOI of ∼10^5^ vg/cell. Full medium changes were performed 5.5 and 24 h after virus exposure. Total RNA was extracted using the RNEasy kit (QIAGEN) 72 h after plating. Library construction and next generation sequencing was performed at Genewiz on three RNA samples (500 ng/sample) in duplicates. The samples were analyzed in two lanes using an Illumina 2000 HI-seq machine that generated between 65 and 91 million reads per sample.

The RNA-seq reads were aligned to the mouse genome mm10 with the RNA computational pipeline on the DNAnexus cloud computing platform comprised of the STAR alignment package and RSEM counting algorithm. Each sample (control, SOX11 wild type, and SOX11^K91A^) had two replicates. The differentially expressed genes were called by using limma-voom in R/BioC on the RNA-seq replicates, and the genes with a FDR < 0.05 and an absolute fold change (FC) > 1.2 were considered for down-stream analysis. The functional enrichment of differentially expressed genes was computed in enrichR, and volcano plots were displayed in R. The RNA-seq data are uploaded in raw format to GEO (accession GSE160627, token wxexuqkmjrcjdgf).

### Statistical analysis

Results are shown as the mean ± SEM of at least three experiments. Data were analyzed by ANOVA with a *post hoc t* test with Tukey correction and/or unpaired *t* test with *p* < 0.05 considered significant.

## Results

### Sox11^K91A^ prevents Sox11 SUMOylation

**Figure 1.**
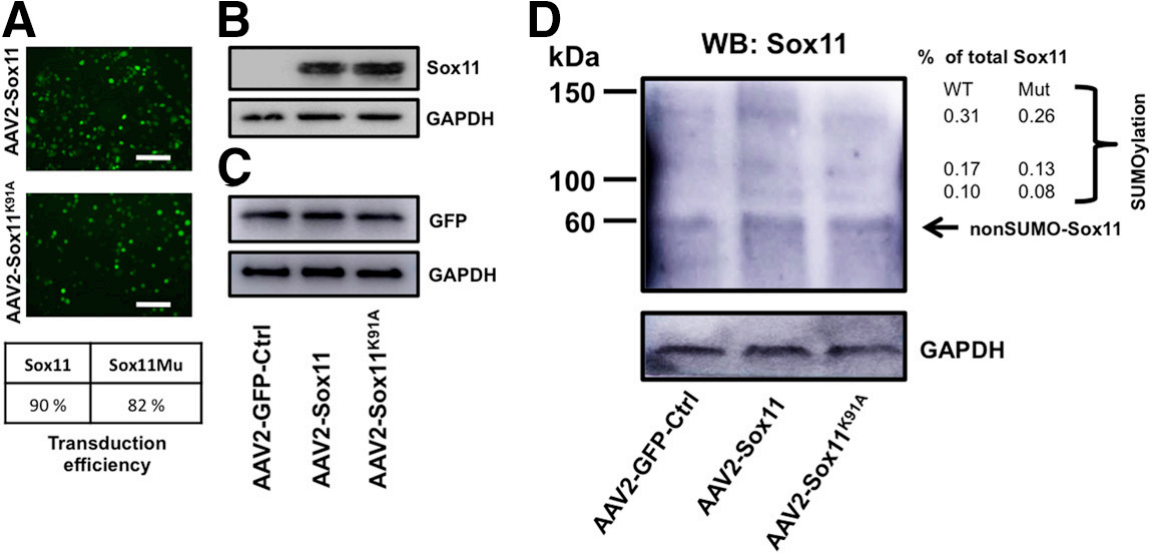
Point mutation of Sox11 lysine 91 to alanine (Sox11^K91A^) attenuates expression of SUMOylated isoforms. ***A***, ***B***, HEK cells (2 × 10^5^ cells/well) and (***C***) hippocampal neurons (10^6^ cells/well) were infected with GFP control, Sox11, and Sox11^K91A^ AAV2 viruses. Fluorescence imaging suggested similar expression levels between Sox11 viral vectors (***A***) and Sox11 and Sox11^K91A^ protein expression were also similar when assayed by Western blotting (***B***, ***C***). ***D***, For SUMOylation detection, P2 RGCs (5 × 10^5^ cells/well) were infected with GFP-conjugated control, Sox11, and Sox11^K91A^ viruses. Quantification of higher molecular weight bands corresponding to total Sox11 protein was detected by Western blotting against Sox11, which detected both endogenous and mutant protein. Scale bar: 100 μm.

To investigate the effects of Sox11 *in vivo*, we first designed AAV-backbone plasmids encoding Sox11 and Sox11^K91A^ and verified protein expression level after 3 d of coculture in HEK 293 cells and hippocampal neurons by immunostaining (Fig. 1*A*) and Western blotting (Fig. 1*B*,*C*). A total of 90% and 82% of cells were transduced by of Sox11 and Sox11^K91A^ AAVs, respectively (Fig. 1*A*). Similar levels of exogenous Sox11 and GFP proteins were detected in HEK 293 cells (Sox11^K91A^ at 1.08-fold expression of Sox 11; Fig. 1*B*) and hippocampal neurons (Sox11 and Sox11^K91A^ at 1.02-fold and 0.98-fold of control AAV; Fig. 1*C*), respectively. To verify whether Sox11^K91A^ is associated with less SUMOylation in primary neurons, we expressed wild-type Sox11 or Sox11^K91A^ in purified RGC cultures and observed fewer higher molecular weight moieties with Sox11^K91A^ than with wild-type Sox11 (Fig. 1*D*). Thus, consistent with our previous findings with a different Sox11 construct (Chang et al., 2017), mutating the K91 residue decreases Sox11 SUMOylation.

### Sox11 and Sox11^K91A^ decrease RGC survival and axon growth *in vitro*

**Figure 2.**
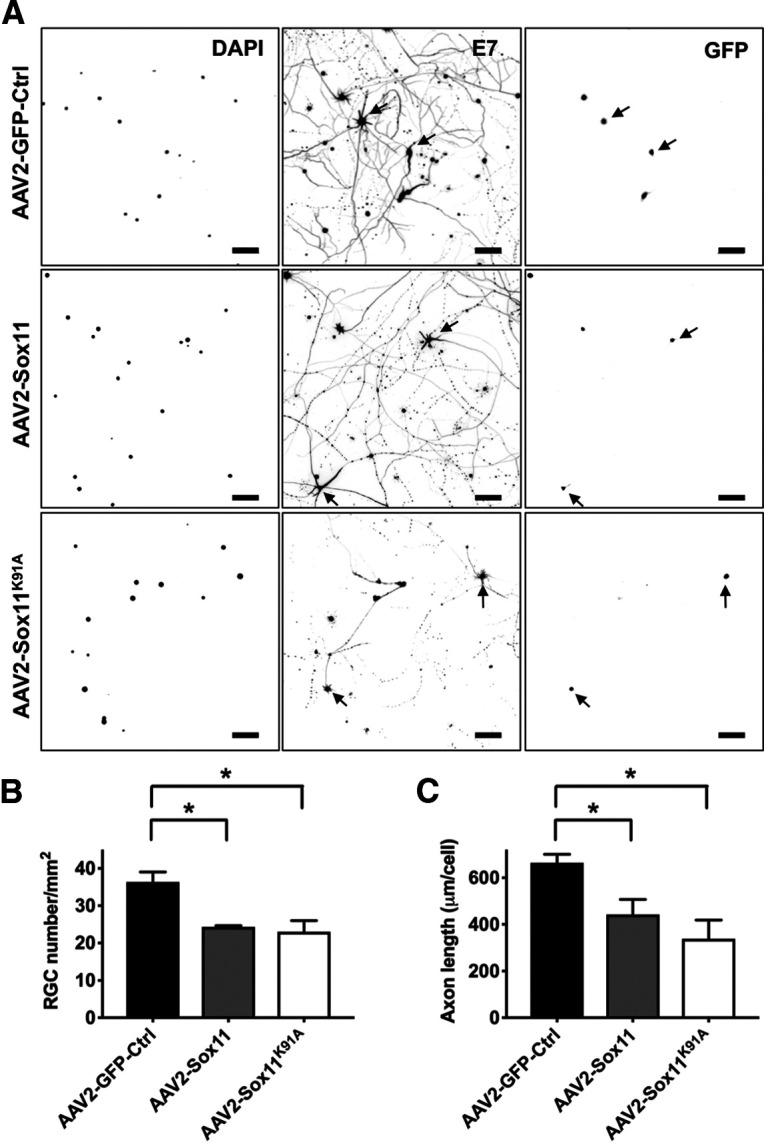
Sox11 and Sox11^K91A^ decrease RGC viability and axon outgrowth *in vitro.* Primary RGCs were transduced with Sox11 or control AAVs as marked, cultured for 3 d, and immunostained for β-III tubulin (E7) and GFP to identified transduced neurons, and counterstained with the nuclear dye DAPI (***A***). Exogenous expression of either Sox11 or Sox11^K91A^ reduced cell survival (***B***) and axon outgrowth (***C***) in primary RGCs (*N* ≥ 3 experimental replicates, *p < 0.05, by one-way ANOVA and *post hoc t* test with Tukey correction; mean ± SEM shown; scale bar: 200 μm).

We next examined Sox11 versus Sox11^K91A^ effects on RGC survival and axon outgrowth *in vitro* (Fig. 2*A*). Compared with AAV2-GFP-Ctrl, AAV2-Sox11 and AAV2-Sox11^K91A^ significantly reduced RGC viability (Fig. 2*B*) and axon outgrowth (Fig. 2*C*) after 3 d *in vitro*. However, we did not observe significant phenotype differences between AAV2-Sox11-treated and AAV2-Sox11^K91A^-treated groups for either phenotype. We repeated this *in vitro* study at 5-fold lower cell density and found a similar trend (data not shown). Thus, Sox11 reduces RGC survival and axon growth *in vitro*, although these data suggest that point mutation of Sox11 on K91 does not amplify these effects, at least *in vitro*.

### Sox11 ^K91A^ more strongly promotes both RGC death and optic nerve regeneration *in vivo*

**Figure 3.**
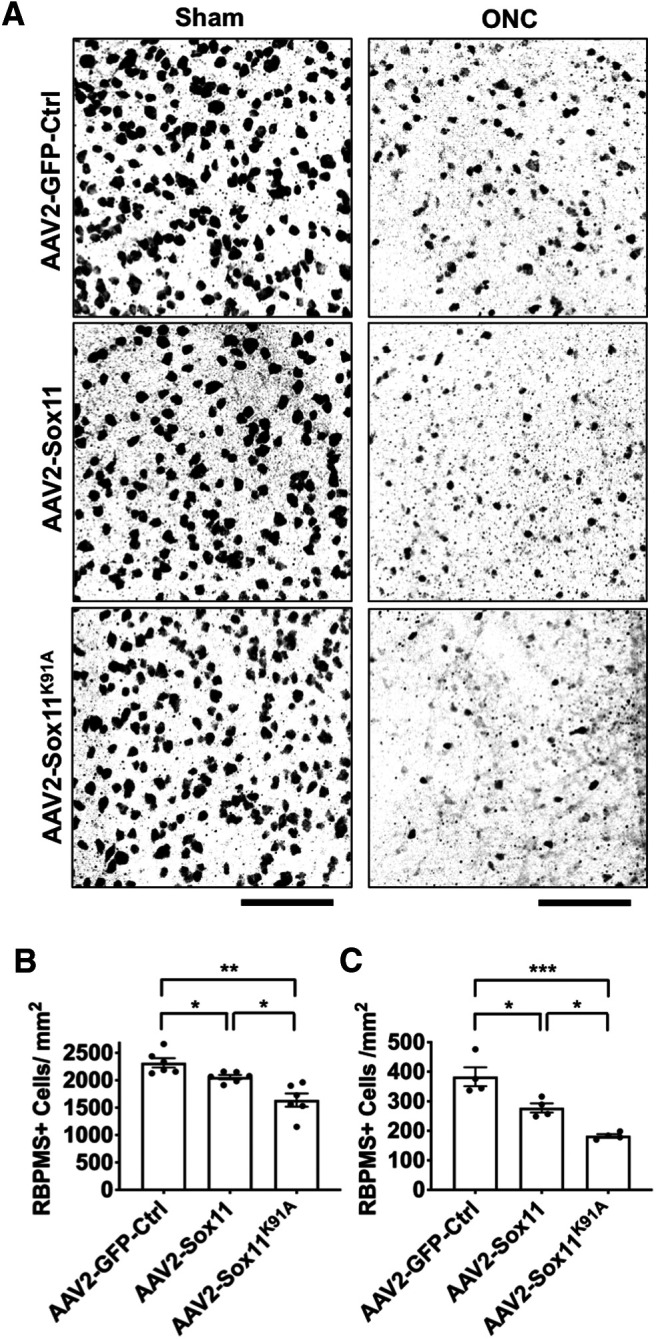
Exogenous Sox11^K91A^ leads to more RGC death than Sox11 *in vivo*. Control AAV2-GFP-Ctrl, AAV2-Sox11, or AAV2-Sox11^K91A^ were injected intravitreally in control eyes and in eyes two weeks before optic nerve crush. Two weeks after injection in controls, or two weeks after optic nerve crush (four weeks after injection), eyes were harvested and retinas flat-mounted and immunostained against RGC-specific marker RBPMS (***A***). Both Sox11 and Sox11^K91A^ significantly increased RGC death in sham (***B***) and optic nerve crush (***C***) eyes, with Sox11^K91A^ showing significantly greater effect than wild-type Sox11 (*N* ≥ 4 experimental replicates, *p < 0.05, **p < 0.01, ***p < 0.001, by one-way ANOVA with *post hoc t* test with Tukey correction; mean ± SEM shown; scale bar: 100 μm).

**Figure 4.**
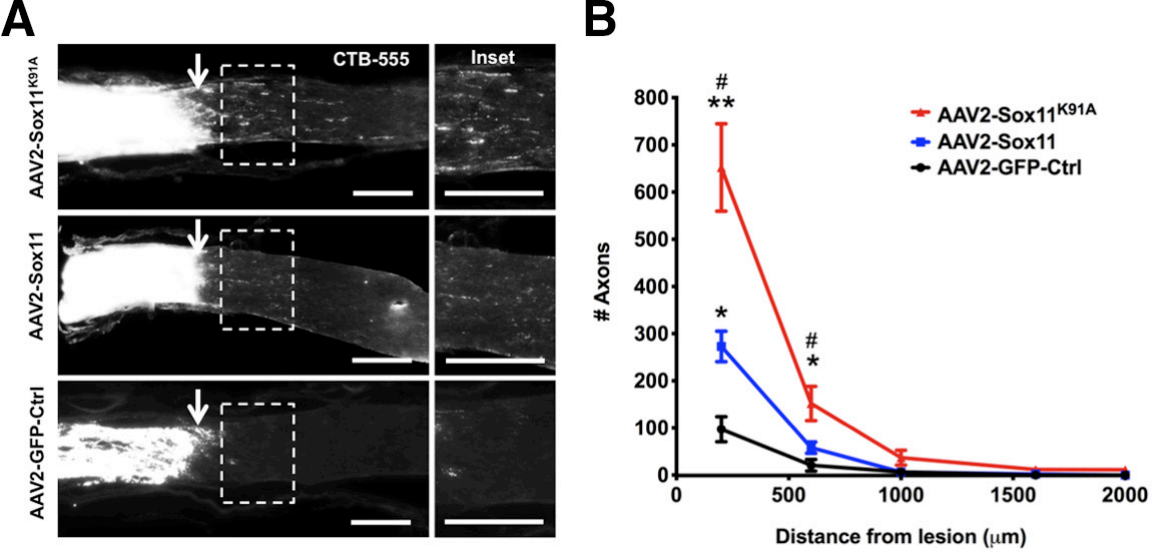
Sox11^K91A^ promotes more axon regeneration than Sox11 *in vivo*. Intravitreal AAV2 injection was performed as in Figure 3. Regenerating axons were visualized by CTB-555 injection anterograde labeling 2 d before euthanasia (***A***). Both Sox11 and Sox11^K91A^ significantly promoted more short distance (200 and 600 μm) axon regeneration than controls, with Sox11^K91A^ showing significantly greater effect than wild-type Sox11 (***B***). The arrows indicate crush sites. *N* ≥ 5 animals per group, * compares with AAV2-GFP-Ctrl group; # compares with AAV2-Sox11 group; *p < 0.05 versus control, **p < 0.01 versus control, #*p* < 0.05 versus AAV2-Sox11, by one-way ANOVA with *post hoc t* test with Tukey correction. Mean ± SEM shown. Scale bar: 200 μm.

To test whether Sox11^K91A^ differs from Sox11 *in vivo*, we intravitreally injected AAVs into normal (uninjured) host eyes, or two weeks before inducing a retrobulbar optic nerve crush injury (Fig. 3*A*). We found that AAV2-Sox11 was associated with increased RGC death *in vivo* in both normal eyes (Fig. 3*B*) or eyes subjected to optic nerve injury (Fig. 3*C*), consistent with our *in vitro* data (Fig. 2) and that from a previous study (Norsworthy et al., 2017). Sox11^K91A^ more strongly promoted RGC death *in vivo* in both conditions (Fig. 3). We also examined axon regeneration in the mouse optic nerve crush model, using anterograde tracing with CTB (Fig. 4*A*). Exogenous Sox11 expression increased the number of CTB^+^ regenerating axons, and Sox11^K91A^ promoted significantly more optic nerve axon regeneration than Sox11 (Fig. 4*B*). Thus, blocking SUMOylation at K91 potentiates axon regeneration in adult RGCs, similar to the potentiation of RGC differentiation during development.

### Sox11 and Sox11 ^K91A^ differently regulate RGC gene expression

**Figure 5.**
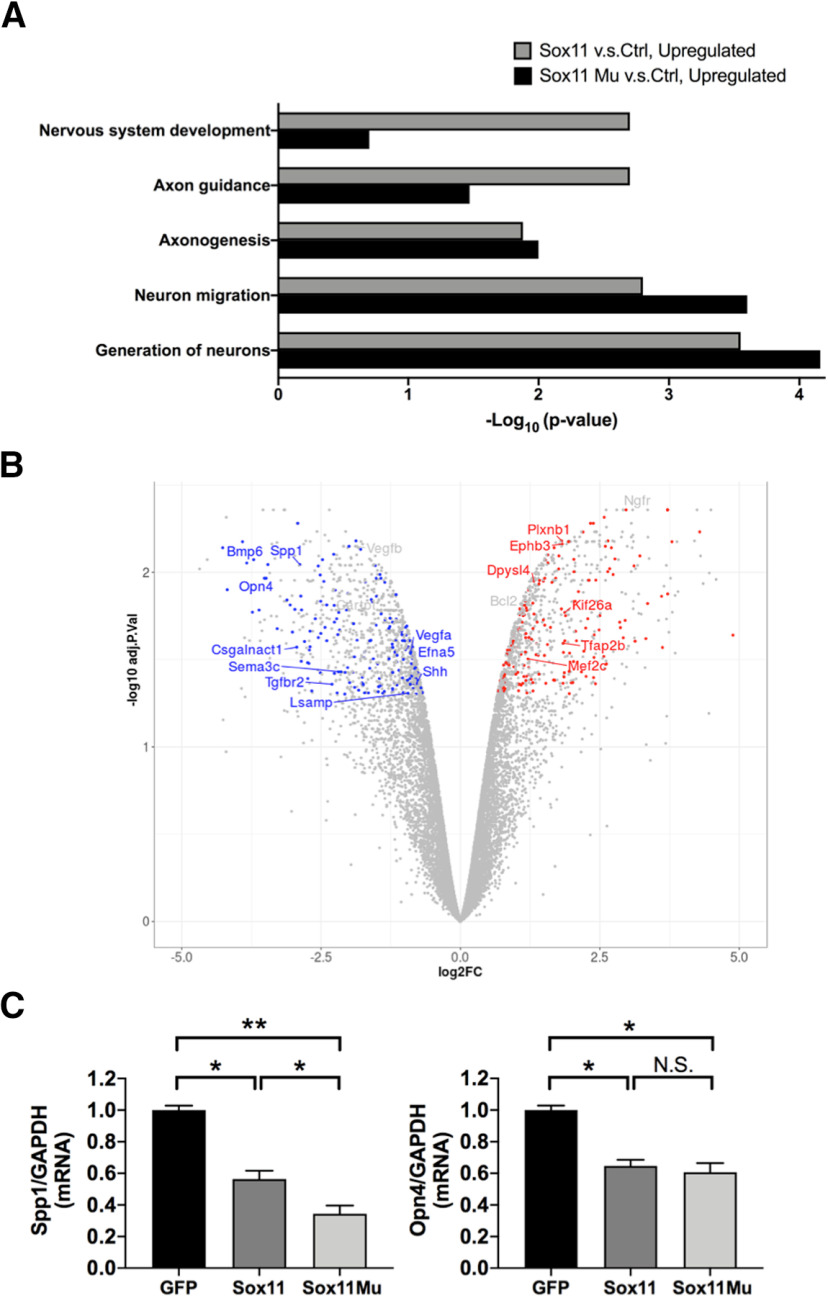
Gene regulation by Sox11 and Sox11^K91A^ in primary RGCs. ***A***, Sox11 and Sox11^K91A^ upregulate several signaling pathways related to neuronal and axon growth. ***B***, A volcano plot highlighting selected genes that are more downregulated by Sox11^K91A^ than by Sox11 (in blue, 173 genes) and selected genes that are more upregulated by Sox11^K91A^ than by Sox11 (in red, 181 genes). ***C***, *Spp1* and *Opn4* were significantly downregulated in Sox11-treated and Sox11^K91A^-treated groups, confirmed by qRT-PCR (*N* = 3, *p < 0.05, **p < 0.01; N.S., no significant difference, by one-way ANOVA with *post hoc t* test with Tukey correction; mean ± SEM shown).

Finally, to explore potential mechanisms by which Sox11 or Sox11^K91A^ differentially affect survival and axon growth, we compared transcriptome changes in RGCs *in vitro* after exogenous expression, using deep sequencing. We first observed that Sox11 and Sox11^K91A^ were upregulated 41-fold and 33-fold (*p* = 5.9E-07 and *p* = 4.4E-03, respectively) in transduced cultures. Exploring gene set enrichment analyses with enrichR, we found that both Sox11 and Sox11^K91A^ upregulated signaling pathways involved in axongenesis, nervous system development, axon guidance, neuron migration and generation of neurons (Fig. 5*A*). Interestingly, Sox11^K91A^ showed stronger effects than Sox11 in genes related to neuronal generation, migration, and axongenesis, but weaker influences on axon guidance and nervous system development in the nucleus. These data suggest the hypothesis that Sox11 in nucleus versus SUMOylated Sox11 in the cytoplasm may regulate neuronal and axonal physiology by different mechanisms. We also detected differentially expressed genes associated with RGC subtype specificity and observed for example that RGC subtype markers *Opn4* (melanopsin) and *Spp1* were downregulated by Sox11 (Fig. 5*B*). The downregulation of *Opn4* and *Spp1* was further confirmed by qRT-PCR (Fig. 5*C*). This is the first report of the Opn4+ intrinsically photosensitive (ip)RGC subtype being influenced by Sox11. Sox11^K91A^ showed a stronger effect than Sox11 on *Spp1* but not *Opn4*, suggesting different susceptibilities of RGC subtypes to Sox11^K91A^.

## Discussion

Taken together, these data indicate three important findings. First, our data reveal that overexpression of a non-SUMOylatable point mutant of Sox11 shows stronger influences than WT Sox11 on decreasing RGC survival but promoting axon regeneration. These data extend our previous study that deSUMOlyated Sox11 translocates into the nucleus to more potently regulate RGC differentiation (Chang et al., 2017). SUMO conjugation acts to regulate many transcription factors, including other Sox family proteins (Williams et al., 2020). Ubiquitin-conjugating enzyme 9 (Ubc9) can interact with Sox4 in the nucleus and repress Sox4’s transcriptional activity, even without its SUMO-1 conjugating capability (Pan et al., 2006). Protein inhibitor of activated STAT (PIAS), known as E3 SUMO-protein ligase, is also known to facilitate protein SUMOylation (Johnson and Gupta, 2001). It will be interesting to dissect out which SUMO ligase(s) are responsible for Sox11 SUMOylation, e.g., through an siRNA or CRISPR screen, in future studies.

Surprisingly, both Sox11 and Sox11^K91A^ suppressed axon outgrowth *in vitro* but promoted axon regeneration *in vivo* after optic nerve crush, with Sox11^K91A^ consistently showing strong effects in either direction than WT Sox11. Despite this difference, we found that signaling pathways involved in axongenesis are upregulated by Sox11 and Sox11^K91A^. RGC density in culture strongly affects axon outgrowth (Goldberg et al., 2002), thus a component of this contradictory data could be explained by low cell density in axon outgrowth assays. In addition, the cell-cell interaction with other retinal cell types *in vivo* is absent in the culture *in vitro*. Regarding cell-type specificity, a previous study showed that Sox11 preferentially kills α-RGCs and other RGC subtypes (Norsworthy et al., 2017), and these subtypes may normally extend longer neurites *in vitro*, a hypothesis that could be tested using the various strains of fluorescently labeled RGC subtypes (Hong et al., 2011; Dhande and Huberman, 2014). It is also possible that Sox11 may regulate axon growth in the young RGCs studied in culture by different mechanisms that the adult RGCs studied for axon regeneration *in vivo*.

Second, we identified candidate signaling pathways regulated by Sox11 and Sox11^K91A^. In the RNA-seq analysis, many signaling pathways related to axonal and neuronal physiology were regulated by Sox11 expression. For example, we found that TGFβR2, a receptor upstream of Smad2/3 signaling, was also downregulated by Sox11 (Fig. 5*B*). These data are interesting in light of previous data showing that suppression of Smad2 expression promotes axon growth in an *in vitro* assay (Hannila et al., 2013), and suggest the hypothesis that Sox11 may promote axon regeneration by regulating TGF/Smad signaling, a question to test in future studies. We also detected upregulation of *MEF2C* expression in Sox11-treated RGCs (Fig. 5*B*), consistent with our and others’ data that MEF2 genes promote RGC death in RGCs (Welsbie et al., 2017), and overexpression of MEF2C induces apoptosis in other cells (Bao et al., 2019). On the other hand, expression of anti-apoptosis gene BclII was upregulated (Fig. 5*B*), consistent with a compensatory response against apoptosis.

Finally, our data add to the literature on RGC subtype-specific regulation. The presence of resilient and susceptible populations of RGCs has been identified based on their different ability to survive or regenerate axons following optic nerve injury (Norsworthy et al., 2017; Tran et al., 2019). Based on Sox11’s downregulation of the photopigment gene melanopsin (encoded by *Opn4*), Sox11 may preferentially lead to the death of another RGC subtype, ipRGCs (Berson et al., 2002; Hattar et al., 2002). We also found that Sox11 downregulates *Spp1* gene expression in RGCs, which is consistent with the previous study that Sox11 leads to death of α-RGCs (a subtyped defined in part by *Spp1* expression). Very little is known about how transcription factors like Sox11 may regulate specific subsets of RGCs or their expression of genes like *Spp1* or *Opn4*, presumably leveraging subtype-specific co-factors or epigenomic access. Since E18 RGC culture was used for RNA-seq, it is possible that Sox11 alters some newborn RGCs toward non-ipRGC and non-α-RGC cell fates. Understanding how Sox11 specifically targets α-RGC and ipRGC would be an interesting future direction that may be better served by future experiments leveraging single-cell RNA-seq (scRNA-seq; Tran et al., 2019). Certainly, placing Sox11 into a molecular regulatory pathway and better understanding its effects in differentially regulating survival and axon regeneration in different RGC subtypes could lead to a more potent therapeutic strategy for axon regeneration.

## Synthesis

Reviewing Editor: Eran Perlson, Tel Aviv University Decisions are customarily a result of the Reviewing Editor and the peer reviewers coming together and discussing their recommendations until a consensus is reached. When revisions are invited, a fact-based synthesis statement explaining their decision and outlining what is needed to prepare a revision will be listed below. The following reviewer(s) agreed to reveal their identity: NONE. Note: If this manuscript was transferred from JNeurosci and a decision was made to accept the manuscript without peer review, a brief statement to this effect will instead be what is listed below.

In this paper titled “Post-translational modification of Sox11 regulates RGC survival and axon regeneration”. The authors utilized AAV2-Sox11K91A to attenuate SUMOylation of Sox11 and investigated whether regenerative and neuroprotective effects are affected by post-translation of Sox11. By using primary RGCs in vitro, the authors investigated the levels of Sox11 SUMOylation and the effects of SUMOylation on axon outgrowth and RGC survival/death. The authors also clarified differential regenerative and neuroprotective outcomes between Sox11 and deSUMOylated Sox11 after ONC in vivo. Finally, this paper also identified differential molecules and pathways between Sox11 vs deSUMOylated Sox11 via unbiased RNA sequencing. The authors identify that non-SUMOylatable Sox11 leads to death and suppressed neuronal outgrowth. The same mutant, however, promotes axonal regeneration in vivo, though increasing RGC death at the same time.

Sox11 has been broadly studied in the field of RGC differentiation and axon guidance during development and also axon regeneration. The present work provides some advances in regards to the ability of Sox11 to regulate regeneration. Specifically, the manuscripts highlights the role of Sox11 post-transnational modifications, thus potentially will give a broad impact to the neuroscience and visual science community. However, the manuscript lacks some depth in regards to the mechanism underlying these phenomena.

There are several issues that need to be addressed before the manuscript is suitable for publication.

Major Comments:

1- The data presented is generated comparing wt Sox11 to a mutant. While the comparison is compelling, the authors should use pharmacological inhibition of SUMO ligases (as they suggest in the discussion) to further clarify the mechanism and add an independent confirm of their results on outgrowth and survival.

2- The weakest point of the paper is the opposite effect of the expression of Sox11 and the mutant in vitro and in vivo in regard to outgrowth. Since this is also the focal point of the paper, it is important that the authors address it not only in the discussion, but by further investigating the underlying mechanism.

3- Connected with the previous point. While the authors perform a sequencing analysis, it needs to be validated by further experiments. The authors should select a few candidates and validate them. This might provide some insight as to point 2.

4- Fig1A should be done with RGC cells, since HEK cells are irrelevant to the study. It is important to know the rate of infection in RGC. Also, Fig1A and B should be quantified.

Minor Comments:

1. In Figure 1C, the top western blot is looking at Sox11 at 4 different molecular weights. The top 3 bands for SUMOylation of Sox11 are faint and the bands around 100kDa are especially hard to identify. Additionally, the left side of the blot has a smear that would impact the control readings. The “% of 60kDa Sox11” table that accompanies the western blot is confusing. Would it not be better to show the magnitude of SUMOylated Sox11 out of the total Sox11? This SUMOylation is supposed to limit the amount of Sox11 that enters the nucleus. Would another western blot that shows Sox11 in the nuclear and cytoplasmic fractions be a better measure of this instead?

2. Figure 3 and 4 have no sub-figure labeled. Labeling the sub-figures as (A), (B), (C) might be helpful.

3. In Figure 4, the authors should show more magnified images of CTB-labeled regenerating axons.

4. Figure 5 shows two similar graphs of signaling pathways that have been upregulated in WT Sox11 / Mutant Sox11 vs Control. The results section compares these two graphs to each other. It may be more beneficial to combine these two graphs together as a double bar chart as this would highlight the differences much better.

5. In line 175, it appears that “wildtype Sox11” is missing right before the word “or”.

6. In line 195, “promoted” is misspelled.

7. In line 217, there is a double space between the words “acts” and “to"

8 - Have the authors provided a link to their sequencing data or at list a supplementary excel file summarizing the results? I could not find it.
